# Estimating the Public Health Impact of Rabies

**DOI:** 10.3201/eid1001.020744

**Published:** 2004-01

**Authors:** Paul G. Coleman, Eric M. Fèvre, Sarah Cleaveland

**Affiliations:** * London School of Hygiene and Tropical Medicine, London, England; † Centre for Tropical Veterinary Medicine, University of Edinburgh, Edinburgh, Scotland

**Keywords:** Rabies, DALY, public health

## Abstract

Rabies is a fatal, preventable zoonosis, but it is not effectively controlled throughout much of the developing world. The impetus for control is hampered by a lack of awareness of its true impact. We estimate a disability-adjusted life year (DALY) score for rabies to quantify the disease impact relative to other diseases to set priorities for public health interventions.

Rabies is a fatal disease that is considered a reemerging zoonosis throughout much of the world (*1*,*2*). Rabies satisfies all the World Health Organization (WHO) criteria for diseases that are a priority for control (*3*) and, unlike many other emerging zoonoses (such as dengue and West Nile virus), safe and effective animal and human vaccines are widely available for its prevention and control. Despite this, rabies remains a neglected disease that is poorly controlled throughout much of the developing world, particularly Africa and Asia, where most human rabies deaths occur (*3*,*4*). A major factor in the failure of rabies control is the low level of political commitment, partly arising from a lack of quantitative data on the true public health impact of the disease (*3*) and the cost-effectiveness and cost benefits of controlling it (*5*).

The disability-adjusted life year (DALY) is a standardized, comparative measure of disease impact developed to assess the relative impact of different diseases across different settings and at different stages of economic and public health development (*6*). The DALY is a combination of the years of life lost (YLL) due to premature death and the years of life lived with a disability (YLD). DALYs have been used to organize disease control in the health sector (*7*) because interventions can be prioritized on the basis of their impact in reducing disease and on the cost-effectiveness of the intervention. Most emerging human diseases are zoonotic (*2*); while DALYs have been estimated for some of these, such as leishmaniasis and trypanosomiasis, a DALY score has never been determined for rabies, which has failed to be considered in any of the annual global disease burden estimates made by WHO (*8*).

## Country-Level Estimates

A DALY estimate, which can be used to rank diseases globally, can also be used to prioritize health interventions at a country level. As a result of widespread problems of data quality and underreporting of rabies, a new approach has recently been adopted in Tanzania to estimate human rabies deaths by using a decision-tree method based on the incidence of human dog-bite injuries. Such bites are reported routinely and more reliably than rabies cases themselves *(9)*. Age-specific human rabies incidence figures calculated from detailed data collected in the Mara Region (*9*), northern Tanzania, were extrapolated to provide a country-level rabies DALY estimate of 42,669 for all of Tanzania in 2000 (Table 1).

**Table 1:** Estimates of the DALY impact of human rabies in Tanzania in 2000^a,b^

Age group (y)	Rabies cases
0–4	10,986
5–14	14,504
15–44	13,876
45–59	1,497
60+	1,807
All ages	42,669

^a^DALY, disability-adjusted life year. ^b^The DALY estimates were based on the estimated incidence of human deaths for Tanzania as reported by Cleaveland et al. *(9)*.

This example demonstrates how a country-specific mortality and DALY estimate can be calculated by using quality data collected from a specific study site. Indeed, the same method used to estimate the annual number of human rabies cases (*9*), and thus DALY impact, in Tanzania may be applied across sub-Saharan Africa to estimate the regional level of underreporting relative to officially reported figures. However, care needs to be taken when extrapolating from small-scale studies to regional and national levels. For example, in Tanzania, country-level estimates of human rabies deaths are likely to be affected by regional variations in rabies incidence in different dog populations (which are the main source of human rabies exposures), availability of postexposure treatment, and levels of knowledge about rabies, which will affect the probability of seeking treatment in hospitals. In addition, knowing the scale of DALYs lost due to a single disease in isolation is not helpful to decision makers prioritizing interventions with limited funds. Better country-level estimates for other diseases also need to be determined. However, this study is a first step.

## Global Estimate

We calculated the global DALY for rabies based on annual WHO estimates of 35,000 deaths (*10*) and using a standard method (*6*) to allow comparison with the most recent estimates (*8*) for the diseases identified for the United Nations Development Program/World Bank/WHO Special Programme for Research and Training in Tropical Diseases, known as TDR (*11*). The figure of 35,000 deaths per year may be expressed in terms of DALYs if certain assumptions about the age and sex distribution of rabies patients are made. Data on the age-related exposure to rabies were obtained from Eng et al. (*12*), a detailed study of human rabies in Mexico. Analysis of dog bite injuries showed a ratio of male:female cases of 0.53:0.47. The age distribution of persons bitten was skewed towards the younger ages (median age 9 years, range <1–84 years) a common pattern seen across developing country settings; 60% of cases occurred in the 0- to 12-year age range, 10% in the 13- to 19-year range, and 30% in the >20 age range.

When these age and sex distributions of patients are used, an annual impact of 35,000 human rabies deaths equates to approximately 1.16 million DALYs. This estimated DALY impact is conservative because it considers only the YLL component and does not takes into account YLDs resulting from the illness associated with the trauma of animal bites and postexposure therapy, if available.

A total of 1.16 million DALYs places rabies just behind trachoma, slightly above onchocerciasis, and well above dengue (Table 2). This estimate shows rabies to be an important disease in terms of DALYs if the WHO figures reflect the true public health situation. However, unlike other zoonoses in the DALY ranking system**,** human rabies is fully preventable by disease control aimed at the animal reservoir. All 1.16 million DALYs could, in theory, be averted through veterinary interventions.

**Table 2:** The global DALY scores for rabies and other selected diseases^a,b^

Disease	Total DALYs lost (x 1,000)
Malaria	42,280
Tuberculosis	36,040
Lymphatic filariasis	5,644
Leishmaniasis	2,357
Schistosomiasis	1,760
Trypanosomiasis	1,598
RABIES	1,160
Onchocerciasis	987
Chagas	649
Dengue	653
Leprosy	177

^a^DALY, disability-adjusted life year. ^b^The DALY score for rabies was based on official World Health Organization (WHO) figures *(10)*. The other listed diseases constitute the official DALY score in 2001 for the priority diseases in the TDR (United Nations Development Program/World Bank/WHO Special Programme for Research and Training in Tropical Diseases) portfolio *(8)*.

Although the above DALY figure gives a useful indication of the global DALY for rabies, the true global incidence (and hence DALY) of human rabies is difficult to assess because rabies is often inconsistently reported. For example, the 1996 World Survey of Rabies (*10*,*13*) recorded a total of 33,212 rabies deaths worldwide (of which 30,000 were reported by India), while only 1,326 were reported in 1991 (when India reported only 34) (*13*). Although rabies is known to be grossly underreported in most developing countries, the degree of underreporting is difficult to assess. However, recent studies from Tanzania indicate that human rabies deaths may be up to 100 times higher than officially reported (*9*), with an estimated incidence of human rabies similar to that recorded during active surveillance studies (*14*). More country-level estimates of underreporting, using methods similar to that developed for Tanzania (*9*), need to be conducted to provide more reliable figures of the true global scale of human rabies. However, even if the 35,000 estimated human rabies cases were more than double the true global figure, the DALY impact attributable to rabies would still be comparable to that of dengue fever, which is recognized by TDR as a major public health threat throughout the tropics.

## Conclusions

The value of providing a quantitative estimate of disease impact due to rabies, even with the inaccuracies of existing case data, should not be underestimated. Rabies is often perceived as a rare or insignificant disease of humans in developing countries; this perception has been a major factor hampering the development of disease control initiatives. Furthermore, control of rabies is often seen as the responsibility of veterinary authorities, but demonstration of the public health importance of rabies and the benefits of disease control to the public health authorities (both in terms of DALYs saved and reduced costs of postexposure treatment) will encourage involvement of the health sector in control efforts. Integration of medical and veterinary sectors is likely to be crucial for effective disease control, as shown by the success of recent rabies control programs in Central and South America, where medical authorities have taken a lead role in implementing mass dog vaccination programs (*15*).

 This first estimate of a global DALY score for rabies, together with the Tanzania-specific example, indicates that the disease exerts a considerable public health impact, exceeding other prominent diseases that currently achieve a higher priority for disease control. Furthermore, the human disease effects of rabies could be eliminated through vaccination of animal reservoirs by using technologies and methods that are available and accessible.
